# The phenolics, antioxidant activity and *in vitro* digestion of pomegranate (*Punica granatum* L.) peels: an investigation of steam explosion pre-treatment

**DOI:** 10.3389/fnut.2023.1161970

**Published:** 2023-04-17

**Authors:** Qi Wang, Tinglan Yuan, Xiaohuan Zhu, Gongshuai Song, Danli Wang, Ling Li, Mingquan Huang, Jinyan Gong

**Affiliations:** ^1^Zhejiang Provincial Key Lab for Biological and Chemical Processing Technologies of Farm Product, School of Biological and Chemical Engineering, Zhejiang University of Science and Technology, Hangzhou, Zhejiang, China; ^2^Key Laboratory of Alcoholic Beverages Quality and Safety of China Light Industry, Beijing Technology and Business University, Beijing, China

**Keywords:** pomegranate peel, steam explosion, phenolic compounds, antioxidant activity, *in vitro* digestion

## Abstract

Pomegranate peels, the main byproduct of pomegranate production, are rich in phenolic compounds that are known for their effective antioxidant properties and have vast application prospects. In this study, steam explosion, an environmentally friendly technique, was applied to pretreat pomegranate peels for phenol extraction. We investigated the effects of explosion pressure, duration, and particle size on the content of total and individual phenolics, and antioxidant activity of pomegranate peels before and after *in vitro* digestion. The optimal conditions for a steam explosion for pomegranate peels in terms of total phenol content were a pressure of 1.5 MPa, a maintenance time of 90 s, and a particle size of 40 mesh. Under these conditions, pomegranate peel extract presented a higher yield of total phenols, gallic acid, and ellagic acid. However, it also had a lower content of punicalin and punicalagin, compared to the unexploded peels. There was no improvement in the antioxidant activity of pomegranate peels after the steam explosion. Moreover, the content of total phenol, gallic acid, ellagic acid, punicalin, and punicalagin, as well as the antioxidant activity of pomegranate peels, all increased after gastric digestion. Nevertheless, there was a large variation in the pomegranate peel processed by different pressure, duration, and sieve fractions. Overall, this study demonstrated that steam explosion pre-treatment could be an efficient method for improving the release of phenolics, especially gallic acid, and ellagic acid, from pomegranate peels.

## 1. Introduction

Pomegranate (*Punica granatum* L.), widely cultivated worldwide, is a food that is used in medicines and dietary supplements (1). The total global production of pomegranate was estimated to be around 3.8 million tons in 2017 (2). It is well known that pomegranate contains bioactive phenolic compounds such as tannins, flavonoids, anthocyanins, and organic acids, which are related to health benefits against diseases (3). However, it has been reported that the most valuable phenolic compounds exist mainly in non-edible peels, which are normally processed as the main byproduct of pomegranate production (4). Pomegranate peels exhibit excellent antioxidant activity because of the abundant polyphenols, which are characterized by a high content of ellagitannins and phenolic acids (5, 6). These phenols have been studied for their numerous beneficial effects on health, including antioxidant, anti-inflammatory, antibacterial, and antiproliferative properties (7–9). Thus, pomegranate peels are an excellent source of high-value antioxidants and have a wide application value (10). However, large amounts of peel are discarded as useless residue, which is a waste of raw materials. Although pomegranate peel phenols are becoming increasingly popular, their extraction is yet to be solved (11). Therefore, it is necessary to develop an efficient method for extracting phenolic compounds for the comprehensive use of pomegranate peels.

Steam explosion is a typical physicochemical pre-treatment technology that has been applied to biomass energy conversion. The instantaneous release of high-temperature and high-pressure steam can destroy the cell wall structure, which results in the release of bioactive substances from plants and supports antioxidant activity (12). Appropriate steam explosion pre-treatment can effectively promote the release of polyphenols and improve the extraction rate. Owing to its time-saving, low energy consumption, environmentally friendly nature, and high extraction efficiency, the steam explosion has been conducted for the extraction and pre-treatment of bioactive ingredients. Among them are phenolic compounds from plants, which have shown striking results. Qin et al. used the steam explosion to extract flavonoids from fig leaves and found that the content of flavonoids from the treated samples was 55.9% higher than that in the control group (13). Xie et al. suggested that steam explosion-treated hulless barley exhibited potent antidiabetic effects and antioxidant capacity (14). Wan et al. reported that steam explosion enhanced the phenolic profiles and antioxidant activity in mung beans (15). Our group previously found that steam explosion increases flavone aglycone content and improves the *in vitro* digestion properties of “Hangbaiju” (16, 17). However, steam explosion pre-treatment of pomegranate peels for phenolic compounds has not been studied.

Due to the low utilization rate of pomegranate peels as food waste, this study examined whether steam explosion could enhance phenolic recovery from pomegranate peels. In addition, it examined whether it could affect phenolic compounds and antioxidant activity. Environmentally speaking, their applications in food, pharmaceuticals, and cosmetics would be of substantial significance (18). Effect of steam explosion (explosion pressure and duration, powder size) on phenolic compounds and antioxidant activity in pomegranate peels was investigated. Furthermore, this study provides the first measurement of phenolic compounds and antioxidant activity of pomegranate peels after *in vitro* gastric and intestinal digestion.

## 2. Materials and methods

### 2.1. Materials

Pomegranate peels were provided by Bozhou Kangyiyin Biotechnology Co. Anhui province, China. Once collected, the pomegranate peels were immediately dried and stored at −20°C. The representative external standards, including punicalagin, punicalin, ellagic acid, and gallic acid, were purchased from Chengdu Master Biotechnology Co. (Chengdu, China). α-Amylase (35 U/mg), pepsin from porcine gastric mucosa (250 U/mg protein), and pancreatin from porcine (24 U/mg of lipase) were purchased from Maclin Reagent Co. (Shanghai, China). Chromatographic grade acetonitrile and methanol were purchased from Merck Co. (Germany). Other reagents not otherwise specified were analytical grade and obtained from Sinopharm Chemical Reagent Co. (Shanghai, China).

### 2.2. Steam explosion pre-treatment

The pomegranate peels were pretreated in a steam explosion jar (QBS-80, Henan Hebi Zhengdao Heavy Machinery Factory, Henan, China) according to the method described by Gong et al. (16). To optimize the parameters of steam explosion, different pre-treatment conditions including explosion pressure (0.5, 1.0, 1.5, and 2.0 MPa), duration (30, 60, 90, 120, and 150 s), and material particle size (20, 40, 60, 80, and 100 mesh) were investigated. After treatment the wet pomegranate peel powder was dried at 45°C, ground, and then smashed through 40 mesh sieve, as the control untreated sample. The prepared samples were stored at −20°C for further analysis.

### 2.3. Scanning electron microscopy

The dried pomegranate peel powders were fixed to the conductive adhesive tape and coated with gold-palladium alloy. Then, the morphologies of pomegranate peel samples were observed by a scanning electron microscope (SU1510, Hitachi, Tokyo, Japan) at 1,000 × magnification.

### 2.4. Extraction of the total phenols

The phenolic compounds of pomegranate peels were extracted by reference to Ge et al. (19) with slight modifications. About 0.5 g pomegranate peel powders were dissolved in 25 mL of methanol solution (70%). The mixture was treated by an ultrasonic bath (KQ-500E, Kunshan Ultrasonic Instrument Co., Ltd, China) under 40 kHz at 35°C for 20 min, and then centrifuged with 8 000 × *g* for 15 min after cooled down. The supernatants were pooled and transferred to a 100-mL volumetric flask and made to volume with methanol solution. Finally, the pomegranate peel extracts were filtered through 0.45 μm microfiltration membrane and stored at −20°C for testing.

### 2.5. *In vitro* digestion

The *in vitro* digestion of pomegranate peel samples was carried out according to the standardized protocol and our previous study with slight modifications (17). First, the simulated oral saliva (SOF) (13 mmol/L NaCl, 1.2 mmol/L CaCl_2_, 20 mmol/L KCl, pH 6.8 ± 0.5), simulated gastric fluid (SGF) (50 mmol/L NaCl, 7 mmol/L NaHCO_3_, 1.35 mmol/L CaCl_2_, 15 mmol/L KCl) and simulated intestinal fluid (SIF) (90 mmol/L NaCl, 3 mmol/L CaCl_2_, 8.7 mmol/L KCl) were prepared as described by Minekus et al. (20). Then, 0.5 g of pomegranate peel powder and 10 mL of SOF added with α-amylase (final concentration, 1.3 g/L) were mixed in a conical flask, adjusted the pH to 6.8, and incubated in the shaking incubator at 37°C for 10 min. Next, 20 mL of SGF was added, the pH was adjusted to 1.2 with HCl (1 mmol/L) before the addition of pepsin solution (final concentration, 3.2 g/L). The gastric mixture was incubated in the shaking incubator at 37°C for 60 min. After the gastric digestion, 10 mL of digested sample was collected and added with 20 mL of methanol for further treatment. Meanwhile, another 10 mL of gastric digested sample was added to 20 mL of SIF, the pH was adjusted to 7.0 followed by adding the porcine pancreatin solution (final concentration, 10.0 g/L). The mixture was incubated in the shaking incubator at 37°C for 120 min. After the digestion, 10 mL of the intestinal digested sample was mixed with methanol (20 mL) for enzyme inactivation. The digested samples were filtrated though microfiltration membrane and dried under vacuum freeze and stored at −20°C and for further analysis.

### 2.6. Determination of the total phenols content (TPC)

The TPC of pomegranate peel samples was determined by the classic Folin-Ciocalteu assay (21). Briefly, the reaction solution was prepared by mixing 1 mL of blank methanol solution, or gallic acid standard (0.05 mg/mL, 0, 0.2, 0.4, 0.6, 0.8, 1.0 mL) or sample with 0.4 mL of Folin-Ciocalteu reagent, 1.0 mL of Na_2_CO_3_ aqueous solution (10%) and 5.6 mL distilled water. The mixture was placed in darkness and incubated for 30 min. Then the absorbance was measured at 760 nm on a UV-5500PC spectrophotometer (Metash Instrument, Shanghai, China). The results were expressed as millimoles of gallic acid equivalents per gram of dry pomegranate peel powder (mg GAE/g).

### 2.7. Phenolic compounds analysis by high-performance liquid chromatography (HPLC)

According to the previous method with some modifications (22), the HPLC (Agilent 1260, Japan) equipped with a UV detector was used for the analysis of main phenolic compounds (punicalin, punicalagin, gallic acid and ellagic acid) in pomegranate peel samples. Separation was conducted in a Phenomenex Luna C18 column (250 mm × 4.6 mm, 5 μm, Torrance, CA, USA) using acetonitrile (phase A) and 0.2% phosphoric acid solution (phase B) at a flow rate of 0.8 mL/min. The modified gradient elution was shown in Supplementary Table 1. The column was maintained at 35°C. The detection wavelength was set at 272 nm. The phenolic compounds were identified and quantified with the corresponding external standards. And the resulting data was expressed as milligram of phenolic compound per gram of the dry peel powder.

### 2.8. Antioxidant activity assays

In this study, the antioxidant activity was evaluated by the 2,2-diphenyl-1-picrylhydrazyl (DPPH) radical scavenging, 2,2′-Azinobis-(3-ethylbenzthiazoline-6-sulfonate (ABTS)) radical scavenging and ferric reducing antioxidant powder (FRAP).

#### 2.8.1. DPPH scavenging assay

The DPPH scavenging assay was conducted according to the method described previously with modifications (23). The sample extract (2 mL, diluted with 70% methanol solution), blank methanol solution, or the antioxidant reference standard Trolox (6-hydroxy-2, 5,7,8-tetramethylchroman-2-carboxylic acid, Sigma-Aldrich, St. Louis, CA, USA) was mixed with DPPH solution (2 mL, 0.15 mmol/L). The mixture was incubated under absolute dark for 30 min. Then the absorbance was recorded at 517 nm with a UV-5500PC spectrophotometer.

#### 2.8.2. ABTS scavenging assay

The ABTS scavenging assay was determined based on the reported method with modifications (24). First, K_2_S_2_O_8_ solution (140 mmol/L) and ABTS stock solution (7 mmol/L) were mixed with a volume ratio of 88:5000 to make the ABTS working solution. Then, the diluted ABTS working solution (3.9 mL) was added to the sample extract (0.1 mL), blank methanol solution, or reference Trolox (0.1 mL) and vortexed. The absorbance was measured at 734 nm.

#### 2.8.3. FRAP assay

For FRAP assay, a published method with some modifications was used (25). The FRAP working solution was prepared by mixing acetate solution (0.1 mol/L, pH 3.6), TPTZ solution (10 mmol/L), and FeCl_3_ solution (20 mmol/L) with a ratio of 10:1:1. The sample extract, or blank, or Trolox (1 mL) was mixed with distilled water (4 mL) and FRAR working solution (2 mL) and vortexed in water bath at 37°C for 10 min. The absorbance was measured at 593 nm on the UV spectrophotometer.

The DPPH, ABTS and FRAP values expressed as the milligram of Trolox equivalent in per gram of dry peel powder (mg TE/g).

### 2.9. Statistical analysis

Each sample was tested at least in triplicate. The data was presented as mean ± standard deviation. Statistical analysis of variance followed with Duncan test was carried out using SPSS 22.0. Significant difference was considered as *P* < 0.05.

## 3. Results

### 3.1. Effect of steam explosion pre-treatment on the microstructure of pomegranate peels

**FIGURE 1:**
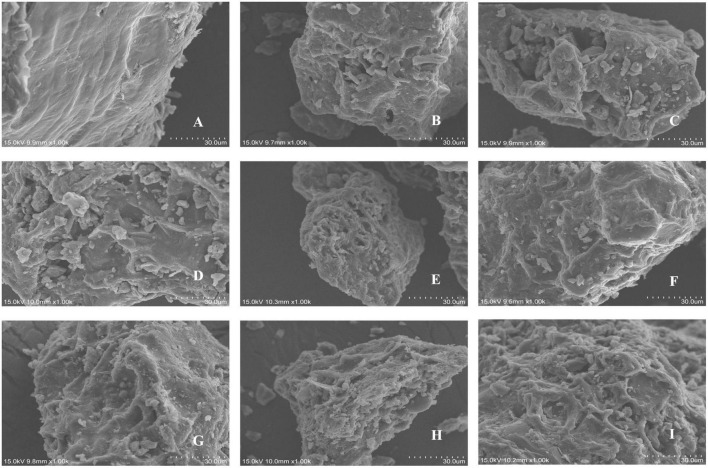
Scanning electron micrographs of different pomegranate peel samples: **(A)** control unexploded sample; **(B)** 0.5 MPa, 90 s; **(C)** 1.0 MPa, 90 s; **(D)** 1.5 MPa, 90 s; **(E)** 2.0 MPa, 90 s; **(F)** 1.5 MPa, 30 s; **(G)** 1.5 MPa, 60 s; **(H)** 1.5 MPa, 120 s; **(I)** 1.5 MPa, 150 s.

The microstructures of the pomegranate peels before and after the steam explosion (Figure 1), treated at different pressures and durations, were observed using scanning electron microscopy. As shown in Figure 1A, before steam explosion pre-treatment, the pomegranate peels had smooth and flat surfaces and relatively complete cells. After steam explosion pre-treatment, the surface of the pomegranate peels became rough and wrinkled. Some ruptures were also observed, indicating that the original tissue state of the pomegranate peel was destroyed. With an increase in explosion pressure and time (Figures 1B–I), large cavities and fragment structures were aggravated. Meanwhile, the viscosity of the powders increased, and the fragments were closely linked. This may be because during the heat treatment process, soluble sugars are degraded from hemicellulose, increasing adhesion (26). Similar results were observed for Adzuki bean (27) and Qingke (28).

### 3.2. Effect of steam explosion pre-treatment on the TPC of pomegranate peels

The changes in TPC in the pomegranate peel under various steam explosion conditions are listed in Table 1. Overall, the TPC of pomegranate peels after the steam explosion (175.02–203.61 mg GAE/g) was higher than that of the unexploded control sample (172.36 mg GAE/g).

**TABLE 1:** Effect of steam explosion (pressure, duration, and particle size) on the total phenol content (TPC) and antioxidant activity of the pomegranate peels.

Samples		TPC (mg GAE/g)	DPPH (mg TE/g)	ABTS (mg TE/g)	FRAP (mg TE/g)
Control		172.36 ± 2.82^aA^	276.65 ± 0.32^AB^	357.20 ± 0.51^b^	527.15 ± 10.45^a^
Steam-explosion pressure (MPa)	0.5	175.02 ± 1.07^ab^	269.44 ± 6.28	328.52 ± 4.43^a^	501.30 ± 12.16^b^
	1	181.50 ± 4.25^b^	267.71 ± 7.34	332.13 ± 14.55^a^	501.39 ± 11.80^b^
	1.5	196.11 ± 3.48^c^	269.40 ± 2.83	333.54 ± 11.36^a^	499.10 ± 3.84^b^
	2	189.15 ± 1.59^c^	272.44 ± 2.54	335.74 ± 2.44^ab^	509.74 ± 5.31^ab^
Steam-explosion duration (s)	30	181.50 ± 1.11^b^	276.56 ± 1.55^AB^	337.30 ± 8.57	524.86 ± 22.54
	60	183.95 ± 2.97^b^	274.55 ± 20.82^AB^	341.87 ± 15.44	527.56 ± 47.40
	90	196.11 ± 3.48^c^	269.40 ± 2.83^a^	333.54 ± 11.36	499.10 ± 3.84
	120	203.61 ± 1.48^c^	290.10 ± 0.65^b^	350.59 ± 3.36	521.31 ± 19.87
	150	196.26 ± 7.39^c^	285.72 ± 3.57^b^	356.44 ± 15.24	535.70 ± 15.13
Particle size (mesh)	20	182.72 ± 2.55*	281.17 ± 4.02	355.00 ± 4.63*	527.22 ± 4.33
	40	196.11 ± 3.48	269.40 ± 2.83	333.54 ± 11.36	499.10 ± 3.84
	60	196.26 ± 2.48	285.68 ± 10.61	354.75 ± 11.62*	523.46 ± 16.63
	80	197.39 ± 11.16	300.78 ± 24.79*	367.06 ± 18.53^#^	562.60 ± 45.92^#^
	100	219.93 ± 6.41^#^	319.79 ± 9.45^#^	407.20 ± 6.22^$^	608.72 ± 43.92 ^$^

Different lowercase letters indicate a statistically significant difference in samples with different pressure (*P* < 0.05). Different uppercase letters indicate a statistically significant difference in samples with different duration (*P* < 0.05). Different symbols indicate a statistically significant difference from the 40-mesh sample, *P < 0.05, ^#^*P* < 0.01, ^$^*P* < 0.001.

The TPC of pomegranate peels increased from 175.02 to 196.11 mg GAE/g as the explosion pressure increased from 0.5 to 1.5 MPa. However, when the pressure was increased to 2 MPa, the TPC decreased slightly (189.15 mg GAE/g). Therefore, 1.5 MPa was chosen as the subsequent steam-explosion pressure. As for duration, when the maintenance time was extended from 30 to 90 s, the TPC increased from 181.50 to 196.11 mg GAE/g. As the time increased to 120 and 150 s, the TPC of pomegranate peel showed a less pronounced increase (203.61 and 196.26 mg GAE/g). Considering the energy consumption, the appropriate duration was fixed at 90 s. When pomegranate peels were exploded at 1.5 MPa for 90 s, with the decrease of particle sizes from 20 to 100 mesh, the TPC increased, with no significant differences among the 40-, 60- and 80-mesh samples (*p* > 0.05). Although the smallest pomegranate peels (100-mesh) had the highest TPC (219.93 mg GAE/g), during the steam explosion process, the occurrence of adhesion between samples led to the powders being heavily linked together. Accordingly, 40-mesh pomegranate peels are appropriate for use in actual operations.

### 3.3. Effect of steam explosion pre-treatment on the phenolic compounds of pomegranate peels

In this study, four main phenols, punicalin, punicalagin, gallic acid, and ellagic acid, were identified. As shown in Table 2, after steam explosion pre-treatment, pomegranate peel samples had more gallic and ellagic acid and less punicalin and punicalagin than the unexploded samples.

**TABLE 2:** Effect of steam explosion (pressure, duration, and particle size) on the phenolic compounds of the pomegranate peels.

Samples		Gallic acid (mg/g)	Punicalin (mg/g)	Punicalagin (mg/g)	Ellagic acid (mg/g)
Control		3.24 ± 0.18^aA^	67.86 ± 4.84^cC^	82.37 ± 7.77^bC^	18.65 ± 1.25^aA^
Steam-explosion pressure (MPa)	0.5	4.91 ± 0.19^b^	24.50 ± 3.95^b^	35.84 ± 5.40^a^	20.74 ± 0.55^a^
	1	4.57 ± 0.47^ab^	15.49 ± 0.23^a^	33.62 ± 5.83^a^	19.36 ± 1.13^a^
	1.5	5.39 ± 0.02^b^	17.50 ± 2.42^ab^	29.35 ± 3.37^a^	25.17 ± 0.72^b^
	2	5.87 ± 1.22^b^	13.41 ± 0.24^a^	33.93 ± 3.57^a^	22.63 ± 3.20^ab^
Steam-explosion duration (s)	30	4.02 ± 0.05^AB^	16.06 ± 2.02^a^	43.68 ± 3.56^b^	17.98 ± 0.39^a^
	60	8.85 ± 1.20^CD^	26.54 ± 5.63^b^	22.32 ± 3.42^a^	26.25 ± 1.40^b^
	90	5.39 ± 0.02^b^	17.50 ± 2.42^a^	29.35 ± 3.37^a^	25.17 ± 0.72^b^
	120	9.74 ± 0.23^D^	16.33 ± 1.34^a^	22.69 ± 2.55^a^	30.63 ± 0.88^C^
	150	7.44 ± 1.13^C^	17.21 ± 3.75^a^	29.33 ± 3.72^a^	28.29 ± 2.70^BC^
Particle size (mesh)	20	5.14 ± 0.20	18.99 ± 6.73	28.39 ± 6.23	22.86 ± 0.63
	40	5.39 ± 0.02	17.50 ± 2.42	29.35 ± 3.37	25.17 ± 0.71
	60	5.96 ± 0.36	34.87 ± 3.80*	31.39 ± 4.37	27.45 ± 0.39
	80	7.55 ± 0.42^#^	12.70 ± 0.95	17.88 ± 2.85	32.37 ± 1.51^$^
	100	5.97 ± 0.53	26.07 ± 7.40	36.37 ± 7.63	30.55 ± 1.48^#^

Different lowercase letters indicate a statistically significant difference in samples with different pressure (*P* < 0.05). Different uppercase letters indicate a statistically significant difference in samples with different duration (*P* < 0.05). Different symbols indicate a statistically significant difference from the 40-mesh sample, *P < 0.05, ^#^*P* < 0.01, ^$^*P* < 0.001.

With an increase in pressure, the content of gallic acid and ellagic acid increased, while punicalin tended to decrease, and punicalagin did not change conspicuously. Regarding the effect of duration, ellagic acid increased with the duration of the explosion maintenance time. The highest concentrations of gallic acid were observed at 60 and 120 s. The 30 and 60 s samples contained the highest contents of punicalin and punicalagin among pomegranate peels that exploded at 1.5 MPa at different times. For particle size, the punicalagin content did not change significantly. Punicalin had the highest content in the 60-mesh sample. The gallic acid and ellagic acid contents first increased and then decreased with decreasing particle size, reaching their highest values in the 80- and 60-mesh samples, respectively. These results suggest that a suitable sieve fraction can enhance the extraction yield of gallic acid and ellagic acid.

### 3.4. Effect of steam explosion pre-treatment on the antioxidant activity of pomegranate peels

The results of the antioxidant activity evaluation of the pomegranate peel are shown in Table 1. There were no significant differences in the DPPH, ABTS, and FRAP values of the pomegranate peel with increasing explosion pressure. This suggests that increased pressure did not enhance the antioxidant activity of the peels. Additionally, the DPPH, ABTS, and FRAP of pomegranate peels pre-treated under different explosion pressures (0.5–2.0 MPa) were weaker than those of the unexploded samples.

The extension of the explosion time improved the DPPH free radical scavenging ability of the pomegranate peels. When the duration was extended from 30 to 150 s, DPPH increased from 276.56 to 290.10 mg TE/g. As a result, the values obtained from 120 to 150 s samples were higher than those obtained before steam explosion treatment. ABTS and FRAP assays showed a similar trend with duration, but the differences were not significant.

In terms of particle size effects, the DPPH, ABTS, and FRAP values all showed a trend of increasing gradually with decreasing particle size. This indicates that the antioxidant activity of pomegranate peels could be elevated significantly with a decrease in particle size. For example, the DPPH, ABTS, and FRAP values of 100- mesh pomegranate peel were 1.19, 1.15, and 1.15 times the 20-mesh sample, respectively, which also significantly exceeded those of peels without steam explosion.

### 3.5. Effect of steam explosion on the *in vitro* digestion of pomegranate peels

#### 3.5.1. TPC

**FIGURE 2:**
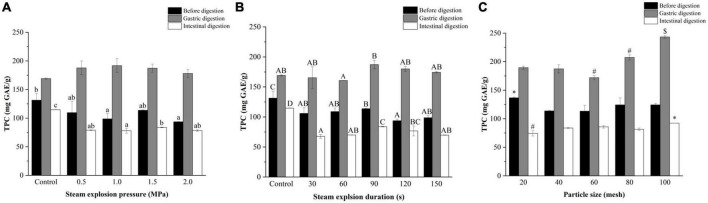
Effects of **(A)** explosion pressure, **(B)** duration, and **(C)** particle size on the total phenol content (TPC) of the pomegranate peels after *in vitro* gastric and intestinal digestion. Different lowercase letters in the same column indicate a statistically significant difference in samples with different pressure (*P* < 0.05). Different uppercase letters in the same column indicate a statistically significant difference in samples with different duration (*P* < 0.05). Different symbols in the same column indicate a statistically significant difference from the 40-mesh sample, *P < 0.05, ^#^*P* < 0.01, ^$^*P* < 0.001.

As shown in Figure 2, the TPC of the pomegranate peels increased after gastric digestion and decreased sharply after intestinal digestion. Notably, the TPC after *in vitro* intestinal digestion was lower compared to before digestion. This trend was particularly evident in the steam explosion samples during digestion.

Steam explosion pressure had little effect on the TPC of pomegranate peels after gastric digestion. After intestinal digestion, peels exploded at 1.5 MPa and had the highest TPC. When the pomegranate peels were pre-treated for different durations, the TPC of the samples after gastric and intestinal digestion increased and then decreased with time, which was the highest in the 90-s sample. Furthermore, the TPC of exploded pomegranate peels increased after *in vitro* digestion, as the particle size decreased. The 100-mesh pomegranate peels after *in vitro* digestion had the most phenols (243.30 mg GAE/g in the gastric phase and 92.11 mg GAE/g in the intestinal phase) and had a higher increment TPC after gastric digestion.

#### 3.5.2. The phenolic compounds

**FIGURE 3:**
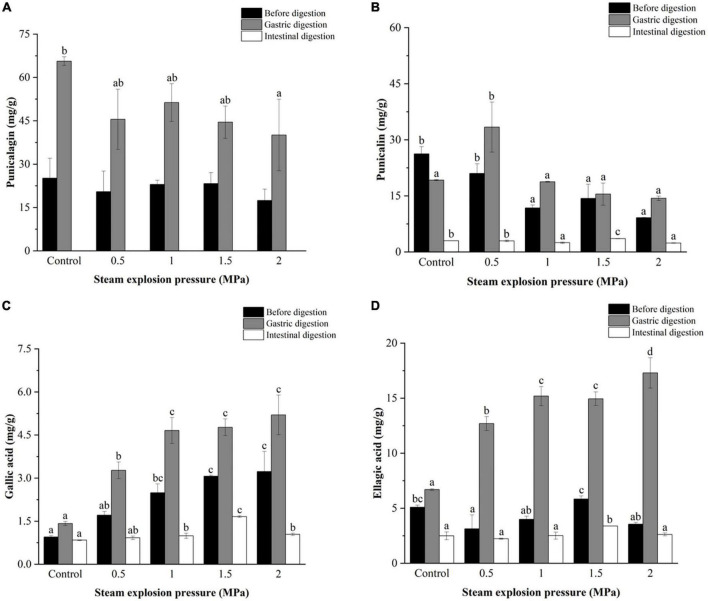
Effects of explosion pressure on the contents of **(A)** punicalagin, **(B)** punicalin, **(C)** gallic acid, and **(D)** ellagic acid in pomegranate peels after *in vitro* gastric and intestinal digestion. Different lowercase letters in the same column indicate a statistically significant difference in samples with different pressure (*P* < 0.05). Different lowercase letters in the same column indicate a statistically significant difference in samples with different duration (*P* < 0.05).

**FIGURE 4:**
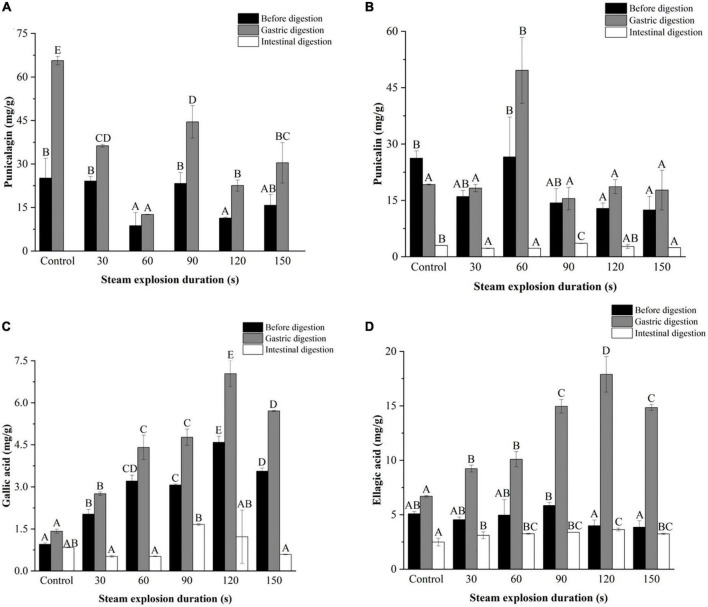
Effects of explosion duration on the contents of **(A)** punicalagin, **(B)** punicalin, **(C)** gallic acid, and **(D)** ellagic acid in pomegranate peels after *in vitro* gastric and intestinal digestion. Different uppercase letters in the same column indicate a statistically significant difference in samples with different pressure (*P* < 0.05). Different uppercase letters in the same column indicate a statistically significant difference in samples with different duration (*P* < 0.05).

**FIGURE 5:**
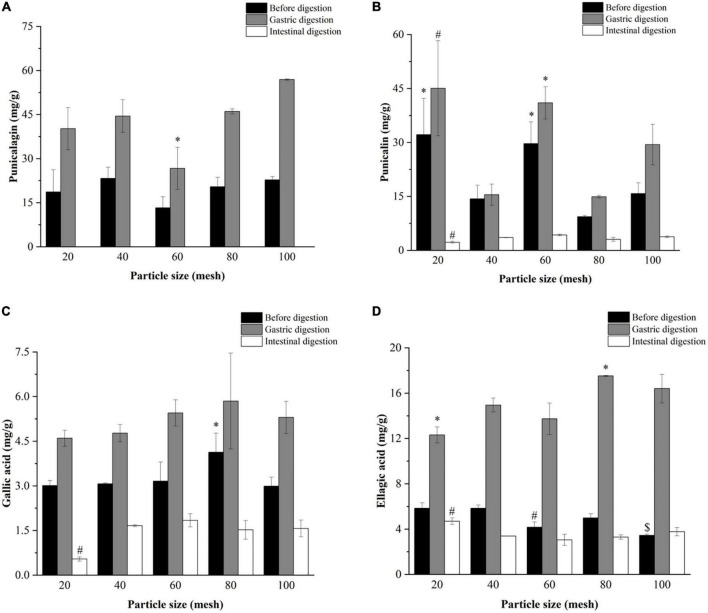
Effects of particle size on the contents of **(A)** punicalagin, **(B)** punicalin, **(C)** gallic acid, and **(D)** ellagic acid in pomegranate peels after *in vitro* gastric and intestinal digestion. Different symbols in the same column indicate a statistically significant difference from the 40-mesh sample, *P < 0.05, ^#^*P* < 0.01, ^$^*P* < 0.001.

Figures 3–5 further illustrate the changes in the contents of gallic acid, punicalin, punicalagin, and ellagic acid in pomegranate peels with steam explosion pre-treatment during the *in vitro* digestion process. The overall trend was that the four phenolic compounds in pomegranate peels significantly increased after gastric digestion and then decreased after intestinal digestion. As a result, punicalagin contents became undetectable after intestinal digestion.

After gastric digestion, the contents of gallic acid and ellagic acid released from pomegranate peels increased gradually with increasing pressure (Figures 3C, D), whereas the contents of punicalin and punicalagin decreased (Figures 3A, B). After intestinal digestion, the contents of gallic acid, punicalin, and ellagic acid increased initially and then decreased as pressure increased. The highest contents (1.66, 3.57, and 3.38 mg/g, respectively) were detected in 1.5 MPa samples.

As shown in Figure 4, the contents of gallic acid and ellagic acid in the gastric and intestinal digests increased from 30 to 120 s but decreased at 150 s. The punicalin and punicalagin contents fluctuated over time. After gastric digestion, the highest contents of punicalagin and punicalin were obtained in pomegranate peels pretreated for 90 and 60 s, respectively. In the end, the highest punicalin content was obtained in pomegranate peels pretreated for 90 s after intestinal digestion.

According to Figure 5, after gastric digestion, gallic acid content remained relatively unchanged after pomegranate peel particle size decreased. However, the content of ellagic acid increased and then decreased in the pomegranate peels. Meanwhile, the punicalin and punicalagin contents fluctuated irregularly with the change in particle size. After intestinal digestion, the contents of gallic acid and punicalin in the 20-mesh pomegranate peels were lower than those of the other-sized samples, whereas ellagic acid showed the opposite result. There were no significant differences in the contents of gallic acid, ellagic acid, and punicalin among the 40-, 60-, 80-, and 100-mesh pomegranate peels.

#### 3.5.3. Antioxidant activity

**FIGURE 6:**
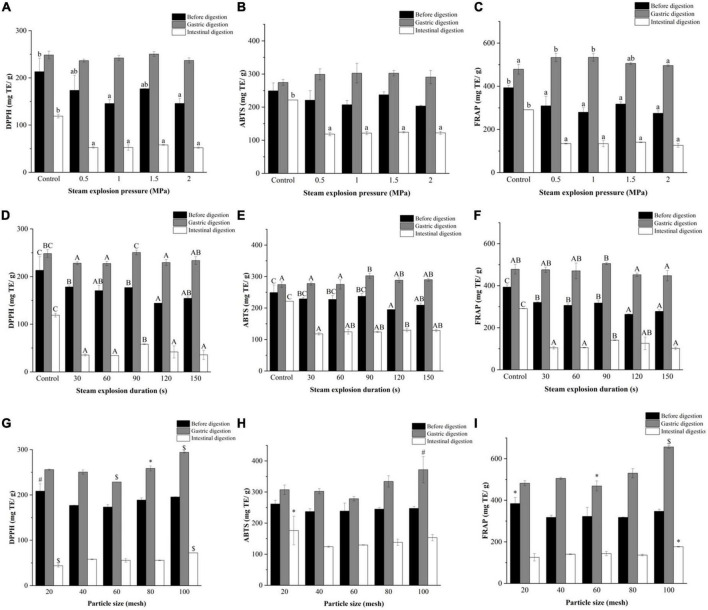
Effects of **(A–C)** explosion pressure, **(D–F)** duration, and **(G–I)** particle size on the antioxidant activity of pomegranate peels after *in vitro* gastric and intestinal digestion. Different lowercase letters in the same column indicate a statistically significant difference in samples with different pressure (*P* < 0.05). Different uppercase letters in the same column indicate a statistically significant difference in samples with different duration (*P* < 0.05). Different symbols in the same column indicate a statistically significant difference from the 40-mesh sample, *P < 0.05, ^#^*P* < 0.01, ^$^*P* < 0.001.

Figure 6 shows the antioxidant activity (the scavenging ability of DPPH, ABTS, and FRAP) of pomegranate peels during *in vitro* digestion. The DPPH, ABTS, and FRAP values of pomegranate peels after gastric digestion were significantly enhanced, whereas they were significantly decreased after intestinal digestion. The DPPH, ABTS, and FRAP values of the unexploded pomegranate peels were similar to or even lower than those of the steam-exploded samples after gastric digestion. However, after intestinal digestion, the three values for the exploded pomegranate peels (250.49, 302.36, and 505.68 mg TE/g) decreased considerably in comparison with the unexploded samples (58.01, 124.07, and 140.54 mg TE/g).

In general, the antioxidant activity of DPPH and ABTS of pomegranate peels after *in vitro* digestion did not change significantly with an increase in explosion pressure. For the duration, the three antioxidant assay values of pomegranate peels after gastric digestion increased initially and then decreased over time. Additionally, the highest values were observed in the 90 s exploded sample. After intestinal digestion, DPPH and FRAP showed the same trend as that after gastric digestion, whereas ABTS did not change significantly over time. As for particle size, the three values showed a relatively consistent upward trend due to the decrease in particle size, and the antioxidant activity in the 100-mesh peels after gastric and intestinal digestion was the strongest.

## 4. Discussion

The steam explosion has been shown to increase the release of phenolics in plants and enhance their antioxidant activity (12, 29). The objective of this study was to identify the effects of steam explosion on the phenolic compounds and antioxidant activity of pomegranate peels. In this study, we found that TPC extracted from pomegranate peels, with the same parameters, increased significantly after steam explosion pre-treatment. Without taking into account the unavoidable differences in samples and experimental conditions, the TPC content of the peel dry weight in this study was higher than the average 26.6 mg GAE/g reported by Masci et al. (6), and 11.8–20.5 mg GAE/g in the study of Rababah et al. (30). Moreover, it was comparable to the ultrasonic-assisted extraction of pomegranate peel phenolics (86.7 mg GAE/g dry weight) (11). Several studies have shown that steam explosion could facilitate the release of phenols from cereal matrices, fruits, and vegetables (12, 29, 31, 32). However, an inverse result was observed in a previous study by Chen et al. (33), who reported that steam explosion decreased the total amount of phenols in soybean seed coats. Therefore, phenols released from plants by the steam explosion might be degraded or transformed, depending on the matrix involved (12).

Furthermore, the explosion pressure (or temperature), duration, and particle size of the matrix are the main factors influencing the extraction of plant phenolics during the steam explosion process. Owing to the high energy density generated by steam explosion, the intercellular mass of the plant loosens, the complex structure is destroyed, and active ingredients are easily dissolved (34). Simultaneously, the breakdown of some sensitive phenolic compounds occurs (17). A balanced state with the dissolution and degradation of phenolic compounds in pomegranate peels through steam explosion pre-treatment was at 1.5 MPa for 90 s. In theory, smaller particles have a larger contact area, which causes more phenols to dissolve; however, they may be easily heated (17, 35). In this study, due to the degradation of hemicellulose and an increase in viscosity (26), some residues of small particle size (80-, 100-mesh) in the material bin were difficult to obtain, resulting in a decrease in extraction efficiency (36). Thus, from the viewpoint of TPC, the optimal conditions for a steam explosion of pomegranate peels were 1.5 MPa, 90 s, and 40 mesh.

By analyzing the changes in the phenolic compounds of pomegranate peels after steam explosion pre-treatment, it was demonstrated that steam explosion could generate shear forces to break the chemical bonds of phenolics, including glycosidic bonds and hydrogen bonds (31). Meanwhile, the steam explosion might facilitate hydrolysis rather than the dissolution of punicalin and punicalagin from pomegranate peels. This might result in the decrease of the two ellagitannins following pre-treatment by steam explosion. Under certain conditions, such as acid, alkali, or high temperature, punicalagin can be decomposed into ellagic acid and punicalin, which can continue to be decomposed into gallic acid (37). This may explain the increased content of gallic acid and ellagic acid, which involved their dissolution from pomegranate peels facilitated by steam explosion and the conversion of punicalin and punicalagin. Interestingly, higher pressure resulted in a decrease in punicalin and an increase in ellagic acid, but no change in gallic acid and punicalagin. This indicates that pressure can facilitate the generation of ellagic acid. In addition, for ellagic acid and gallic acid, the optimal steam explosion conditions were 1.5 MPa for 120 s, whereas steam explosion was not suitable for the extraction of punicalin and punicalagin from pomegranate peels.

It has been known that phenolic compounds play a vital role in the antioxidant activity of pomegranate peels (38). Phenols were readily released from pomegranate peels through steam explosion pre-treatment, resulting in improved antioxidant activity. Notably, exploded pomegranate peels with small particles (100 mesh) displayed excellent antioxidant activity. A possible reason for this is the high TPC (6). However, although the TPC in pomegranate peels increased with increasing explosion pressure and duration, antioxidant activity was not remarkably enhanced, as expected. This may be because the steam explosion changed the composition of the phenolic compounds. Structural differences and spatial configuration of substituents can result in different biological activities (3). Each molecule of punicalagin, punicalin, gallic acid, and ellagic acid contains 16, 10, 4, and 3 phenolic hydroxyl groups (see Supplementary Figure 1), respectively, and their antioxidant capacity is successively reduced (39). Therefore, the slight improvement in antioxidant activity might be related to the decrease in the punicalagin and punicalin content of pomegranate peels with increasing steam explosion pressure and duration. Gil et al. showed that punicalagin was responsible for the antioxidant activity of pomegranate juice (40). The structural identification of phenolic compounds in pomegranate peels with steam explosion pre-treatment and their relationship with antioxidant activity need to be confirmed in further research.

Considering that pomegranate peel powder was used in the digestion experiment instead of the pomegranate peel extract, the dissolution and hydrolysis of phenolic substances occurred simultaneously during the process of digestion. Phenolic compounds are thought to be more reactive at an acidic pH in the gastric phase and less reactive in the intestinal phase (41). Therefore, in the intestinal digestion phase, the phenolic substances released in the gastric digestion phase were hydrolyzed in large quantities. Furthermore, the hydrolyzed amount far exceeded the continued dissolved amount, resulting in a decline in TPC after intestinal digestion. The increase in TPC in the exploded peels was much higher than in the unexploded peels after gastric digestion, suggesting that steam explosion contributed to disconnecting the interaction formed between phenols and other compounds (14). In addition, the four phenolic compounds in pomegranate peel were easily degraded or transformed during *in vitro* digestion. Previous *in vivo* studies have reported that ellagitannins can be hydrolyzed to ellagic acid in the small intestine and cecum (42). The increase in punicalagin after gastric digestion might be caused by continuous dissolution at the stage of gastric digestion. Moreover, the dissolution amount was much larger than the hydrolyzed amount, showing a trend of substantial increase. Nevertheless, in the intestinal digestion phase, the dissolved punicalin and punicalagin in the gastric digestion phase were hydrolyzed, showing a trend of substantial decrease.

**FIGURE 7:**
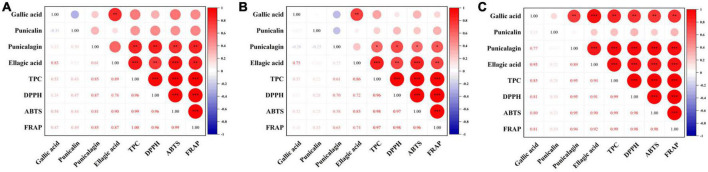
Correlation analysis between phenolic and antioxidant activity of pomegranate peels: **(A)** steam pressure; **(B)** steam duration; **(C)** particle size. *Indicated a significant correlation at 0.05 significant level. **Indicated a strong significant correlation at 0.01 significant level. ***Indicated a strong significant correlation at 0.001 significant level.

Research suggests that higher TPC corresponds to greater antioxidant activity (6). According to our correlation analysis of antioxidant activity and phenolic compounds (Figure 7), DPPH, ABTS, and FRAP of pomegranate peels during *in vitro* digestion were positively correlated with TPC, as well as punicalagin and ellagic acid content. These results imply that the bioaccessibility of specific phenolic compounds should be considered, except for the total phenols in the pomegranate peel processing, which could have influenced the health benefits of the fruit.

## 5. Conclusion

In summary, this study demonstrated that steam explosion is an efficient method for the extraction of phenolics from pomegranate peels. Through steam explosion pre-treatment, the TPC of pomegranate peels increased, whereas antioxidant activity was not significantly enhanced. The phenolic compounds in pomegranate peels could be influenced by steam explosion pre-treatment. This increased the contents of ellagic acid and gallic acid, and decreased the content of punicalin and punicalagin, thereby counteracting the antioxidant activity of phenolic compounds. The appropriate steam explosion conditions for pomegranate peels were pressure 1.5 MPa, duration 90 s and 40-mesh for the extraction of total phenols, and 1.5 Mpa and 120 s for the extraction of ellagic acid and gallic acid. Additionally, the levels of the four phenolic compounds and antioxidant activity increased after gastric digestion and significantly decreased after intestinal digestion. The steam explosion caused the phenolic compounds in the pomegranate peels to be released more rapidly during digestion. Nevertheless, little is known about how transformed molecules and other bioactive compounds contribute to the antioxidant activity of pomegranate peels. Further, the underlying mechanism of how steam explosion alters the interaction between phenols and other compounds present in pomegranate peels needs to be explored in the future.

## Data availability statement

The original contributions presented in this study are included in the article/Supplementary material, further inquiries can be directed to the corresponding authors.

## Author contributions

JG conceived the study. QW and XZ performed the experiments. TY wrote the original manuscript. GS, DW, and LL analyzed the data. TY, JG, and MH reviewed and edited the manuscript. All authors read, discussed, and agreed to the published version of the manuscript.
