# Supplementary material for: The phenolics, antioxidant activity and in vitro digestion of pomegranate (Punica granatum L.) peels: an investigation of steam explosion pre-treatment
Source: Front Nutr. 2023 Apr 17;10:1161970. doi: 10.3389/fnut.2023.1161970 (PMC10149855; doi:10.3389/fnut.2023.1161970)
Supplement: Supplementary file 1 [file Data_Sheet_1.docx]

**Supplementary material**

Figure S1 Structures of phenolic compounds

|  |  |
| --- | --- |
| gallic acid | ellagic acid |
|  | |
| punicalin | |
|  | |
| punicalagin | |

Figure S1 Structures of phenolic compounds

**Table S1 The gradient elution program**

| Time (min) | Phase A (%) | Phase B (%) |
| --- | --- | --- |
| 0 | 2 | 98 |
| 10 | 13 | 87 |
| 20 | 13 | 87 |
| 20.5 | 30 | 70 |
| 30 | 30 | 70 |
| 35 | 2 | 98 |
| 45 | 2 | 98 |
